# Supplementary material for: In situ cryo-ET visualization of mitochondrial depolarization and mitophagic engulfment
Source: bioRxiv. 2025 Mar 25:2025.03.24.645001. Preprint. [Version 1] doi: 10.1101/2025.03.24.645001 (PMC11974748; doi:10.1101/2025.03.24.645001)
Supplement: Supplement 1 [file media-1.pdf]

RESOURCE TYPE	RESOURCE NAME	SOURCE	IDENTIFIER	NEW/REUSE	ADDITIONAL INFORMATION
**Dataset**	structural coordinates	rcsb.org	in progress	NEW	
**Dataset**	density maps	ebi.ac.uk/emdb	in progress	NEW	
**Dataset**	raw cryo-EM data	ebi.ac.uk/empiar	in progress	NEW	
**Dataset**					
**Software/code**	Zeiss ZEN Blue software version 3.7	Carl Zeiss LLC	RRID:SCR_013672	REUSE	
**Software/code**	Arivis Vision4D (ver 4.2.1)	Carl Zeiss LLC	RRID:SCR_018000	REUSE	
**Software/code**	Scipion		RRID:SCR_016738	REUSE	https://scipion.i2pc.es/
**Software/code**	Membrain		https://github.com/CellArchLab/MemBrain-v2	REUSE	
**Software/code**	Dragonfly			REUSE	https://dx.doi.org/10.3791/64435-v
**Software/code**	Pytom model		https://github.com/SBC-Utrecht/PyTom	REUSE	
**Software/code**	AlphaFold3		RRID:SCR_025885	REUSE	
**Software/code**	ImageJ		RRID:SCR_002285	REUSE	https://imagej.net/
**Software/code**	JACoP		RRID: SCR_025164	REUSE	https://imagej.net/plugins/jacop
**Software/code**	GraphPad Prism 9		RRID:SCR_002798	REUSE	
**Software/code**	ISOLDE (ver 1.5)	PMID: 29872003	n/a	REUSE	https://isolde.cimr.cam.ac.uk/
**Software/code**	MotionCor3		https://github.com/czimaginginstitute/MotionCor3	REUSE	
**Software/code**	3DEM		RRID:SCR_016738	REUSE	https://github.com/3dem/relion
**Software/code**	CTFfind 5		https://github.com/GrigorieffLab/ctffind5_manuscript	REUSE	
**Software/code**	AreTomo2		https://github.com/czimaginginstitute/AreTomo2	REUSE	
**Software/code**	ChimeraX (ver 1.5)	PMID: 32881101	RRID:SCR_015872	REUSE	https://www.cgl.ucsf.edu/chimera/
**Software/code**	Napari		RRID:SCR_022765	REUSE	https://www.napari-hub.org/
**Software/code**	Relion 5		https://github.com/3dem/relion	REUSE	
**Software/code**	GraphPad Prism (ver 10.1.0)	Graphstats Technologies		REUSE	http://www.graphpad.com/
**Software/code**	Watershed			REUSE	
**Protocols**	OA treatment and Quantification of Parkin recruitment to mitoch	protocols.io	https://dx.doi.org/10.17504/protocols.io.5qpvoox4dv4o/v1	NEW	
**Protocols**	Transfection	protocols.io	http://dx.doi.org/10.17504/protocols.io.ewov1dr82vr2/v1	NEW	
**Protocols**	Cell culture and cell line generation	protocols.io	https://dx.doi.org/10.17504/protocols.io.81wgbxq2qlpk/v1	REUSE	
**Protocols**	Mitophagy flux assay via in-gel fluorescence	protocols.io	https://dx.doi.org/10.17504/protocols.io.x54v9pwkmg3e/v1	REUSE	
**Protocols**	Lentivirus plasmids generation	protocols.io	https://dx.doi.org/10.17504/protocols.io.yxmvm3z5bl3p/v1	REUSE	
**Protocols**	EM grid seeding and cryo-FIB milling	protocols.io	https://dx.doi.org/10.17504/protocols.io.dm6gpde65gzp/v1	NEW	
**Protocols**	Cryo-electron tomography data acquisition	protocols.io	https://dx.doi.org/10.17504/protocols.io.36wgq6rkklk5/v1	NEW	
**Protocols**	Cryo-electron tomography data processing and model building	protocols.io	https://dx.doi.org/10.17504/protocols.io.36wgq6rkklk5/v1	NEW	
**Protocols**	Live Airyscan microscopy of mitochondrial ultrastructure	protocols.io	in progress		
**Antibody**	/				
**Bacterial strain**	/				
**Virus strain**	/				
**Biological sample**	/				
**Chemical, peptide, or recombinant protein**	Lipofectamine	Thermo Fisher Scientific	REF:11668030	REUSE	
**Chemical, peptide, or recombinant protein**	Opti-MEM	Thermo Fisher Scientific	REF: 31985-070	REUSE	
**Critical commercial assay**	/				
**Experimental model: Cell line**	U-2OS	Cell Culture Facility, UC Berkeley	RRID:SCR_017924	REUSE	
**Experimental model: Cell line**	HEK293T	Cell Culture Facility, UC Berkeley	RRID:CVCL_0063	REUSE	
**Oligonucleotide**	/				
**Recombinant DNA**	mCherry-TOMM20-N-10	AddGene	Plasmid # 55146	REUSE	500ng per dish
**Recombinant DNA**	ATP5F1B-tGFP	OriGene	NM_001686, SKU: RG201638	NEW	250ng per dish
**Recombinant DNA**	pCMV-VSV-G	Addgene	Addgene_8454	REUSE	https://www.addgene.org/8454/
**Recombinant DNA**	pCMVR8.74	Addgene	Addgene_22036	REUSE	https://www.addgene.org/22036/
**Recombinant DNA**	pBS-CMV-gagpol	Addgene	Addgene_35614	REUSE	https://www.addgene.org/35614/
**Recombinant DNA**	pLV-mCherry-Parkin	Addgene	Addgene_237397	NEW	
**Recombinant DNA**	pLV-BFP-mito	Addgene	Addgene_237398	NEW	
**Recombinant DNA**	pMRX-IB-pSu9-HaloTag7-mGFP	Addgene	Addgene_184905	REUSE	https://www.addgene.org/184905/
**Recombinant DNA**	pMK1253	Addgene	Addgene_133058	REUSE	https://www.addgene.org/133058/
**Recombinant DNA**	pBMN-mCherry-Parkin	Addgene	RRID:Addgene_59419	REUSE	https://www.addgene.org/59419/
**Recombinant DNA**	EBFP2-Mito-7	Addgene	Addgene_55248	REUSE	https://www.addgene.org/55248/
