# Supplementary material for: In situ cryo-ET visualization of mitochondrial depolarization and mitophagic engulfment
Source: bioRxiv. 2025 Mar 25:2025.03.24.645001. Preprint. [Version 1] doi: 10.1101/2025.03.24.645001 (PMC11974748; doi:10.1101/2025.03.24.645001)
Supplement: Supplement 7 [file NIHPP2025.03.24.645001v1-supplement-7.pdf]

bioRxiv preprint doi: https://doi.org/10.1101/2025.03.24.645001; this version posted March 25, 2025. The copyright holder for this preprint (which was not certified by peer review) is the author/funder, who has granted bioRxiv a license to display the preprint in perpetuity. It is made available under aCC-BY 4.0 International license.

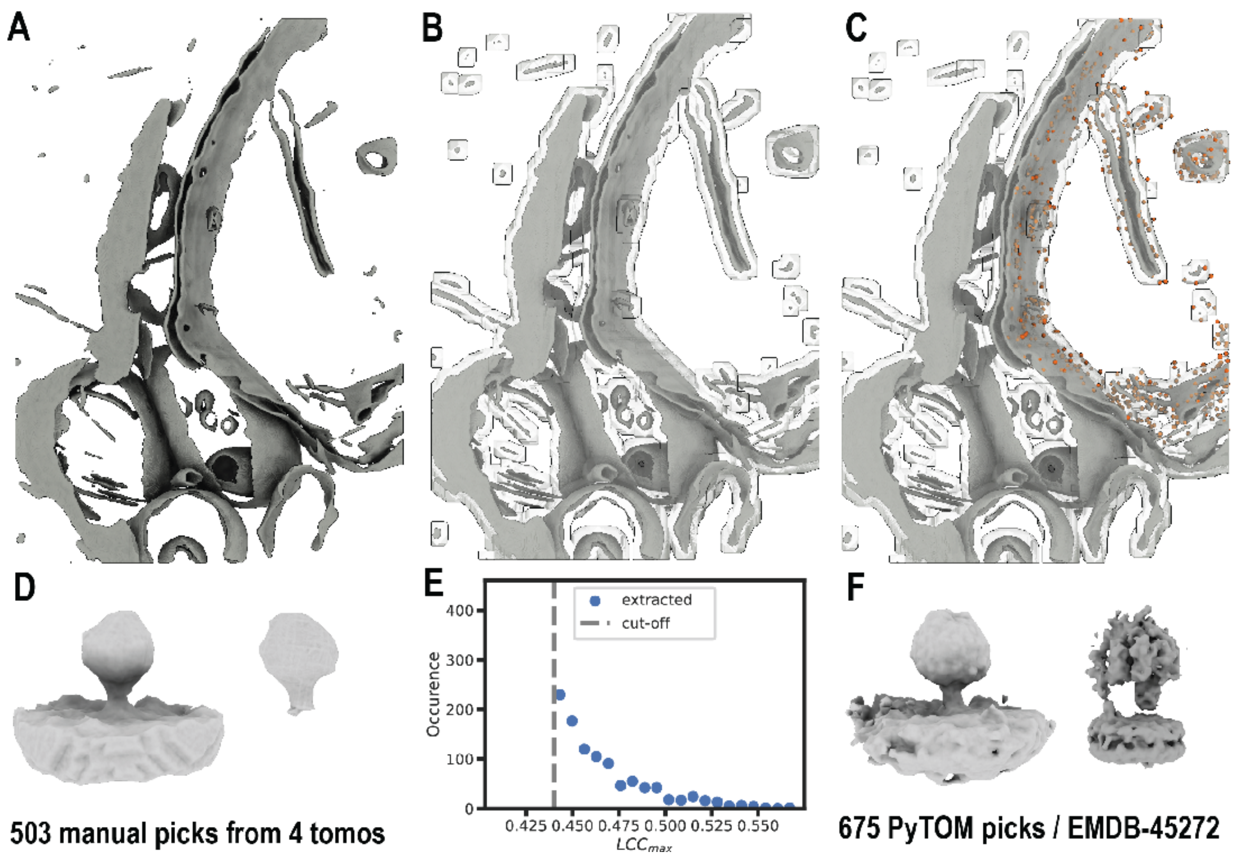

Supplemental Figure 1: Template match picking strategy for ATP synthase. Membranes from tomograms were first segmented to guide the particle picking process (A). A mask to guide template match picking was generated using the membrane segmentation (B). Output of template match picking (C) using the resulting sub-tomogram average from 503 manual picks of 4 tomograms with membrane (left) or without (right) (D). (E) PyTOM parameters used to restrain template matching. Initial sub-tomogram average from 675 template match picks from PyTOM compared to a recently solved density map (EMDB-45272) (F).

bioRxiv preprint doi: https://doi.org/10.1101/2025.03.24.645001; this version posted March 25, 2025. The copyright holder for this preprint (which was not certified by peer review) is the author/funder, who has granted bioRxiv a license to display the preprint in perpetuity. It is made available under aCC-BY 4.0 International license.

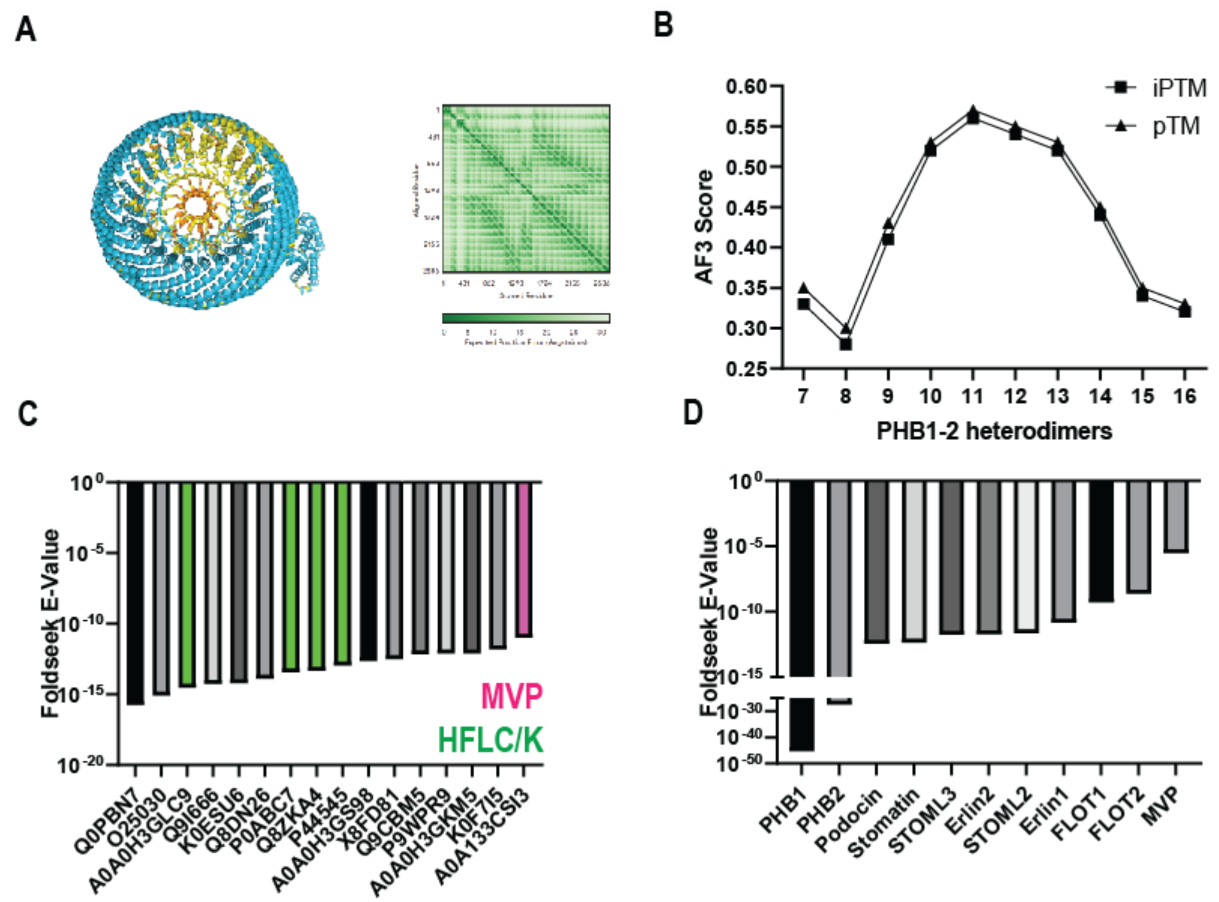

Supplementary Figure 2: Structural analysis and comparison of Prohibitin and the closely related HFLK/C complexes. Alphafold modeling of a heterododecamer Prohibitin 1-2 complex and resultant pLDDT plot from Alphafold 3 with 2 heterodimer copies (A). Alphafold 3 screen using 2 heterodimer copies shows a preferred stoichiometry between 11 and 12 heterodimer copies (B). Foldseek using the structure of human prohibitin shows greater E-Values to bacterial HFLC/K structures than to other closely related mammalian SPFH domain-containing proteins (C-D).

bioRxiv preprint doi: https://doi.org/10.1101/2025.03.24.645001; this version posted March 25, 2025. The copyright holder for this preprint (which was not certified by peer review) is the author/funder, who has granted bioRxiv a license to display the preprint in perpetuity. It is made available under aCC-BY 4.0 International license.

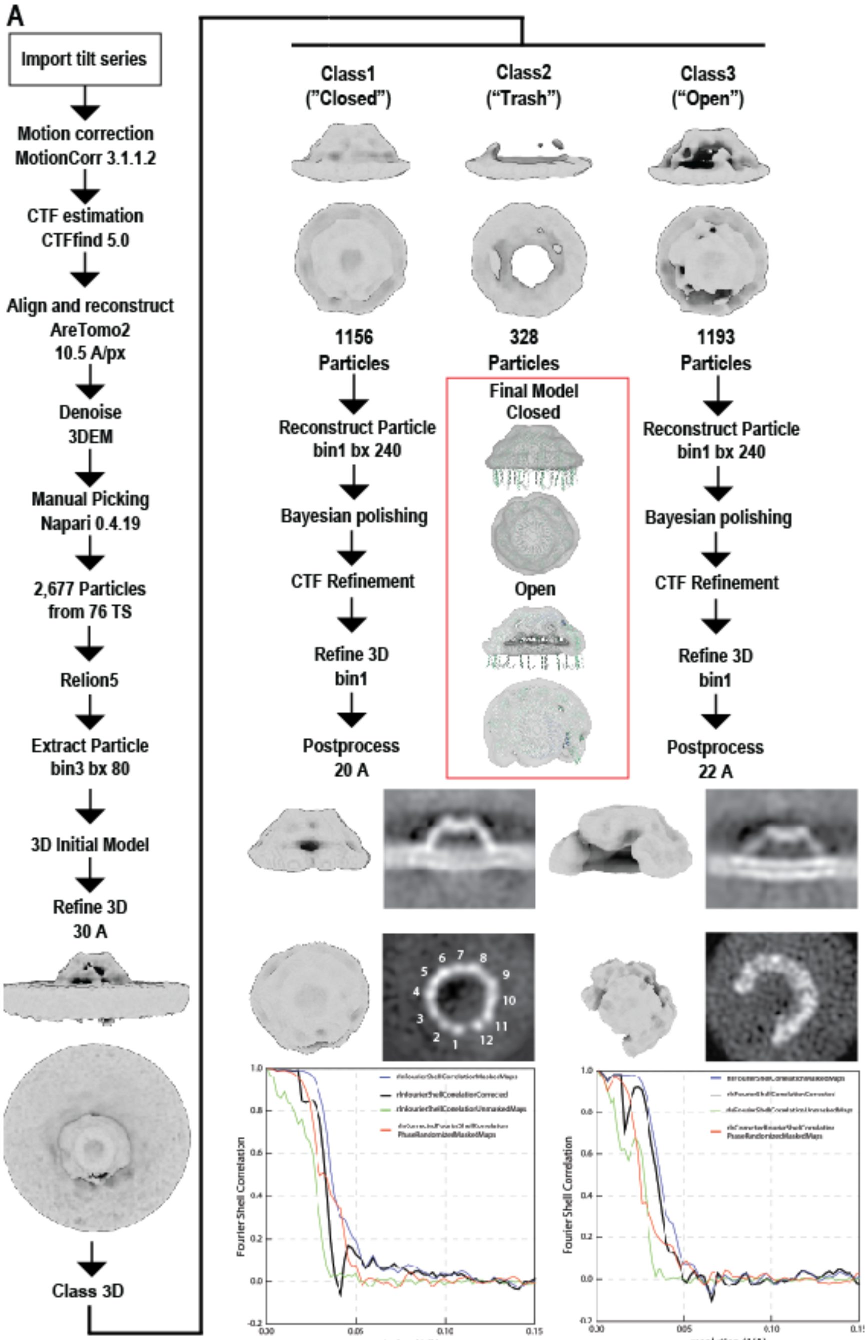

Supplementary Figure 3: Data processing pipeline for sub-tomogram averaging of the prohibitins complex

bioRxiv preprint doi: https://doi.org/10.1101/2025.03.24.645001; this version posted March 25, 2025. The copyright holder for this preprint (which was not certified by peer review) is the author/funder, who has granted bioRxiv a license to display the preprint in perpetuity. It is made available under aCC-BY 4.0 International license.

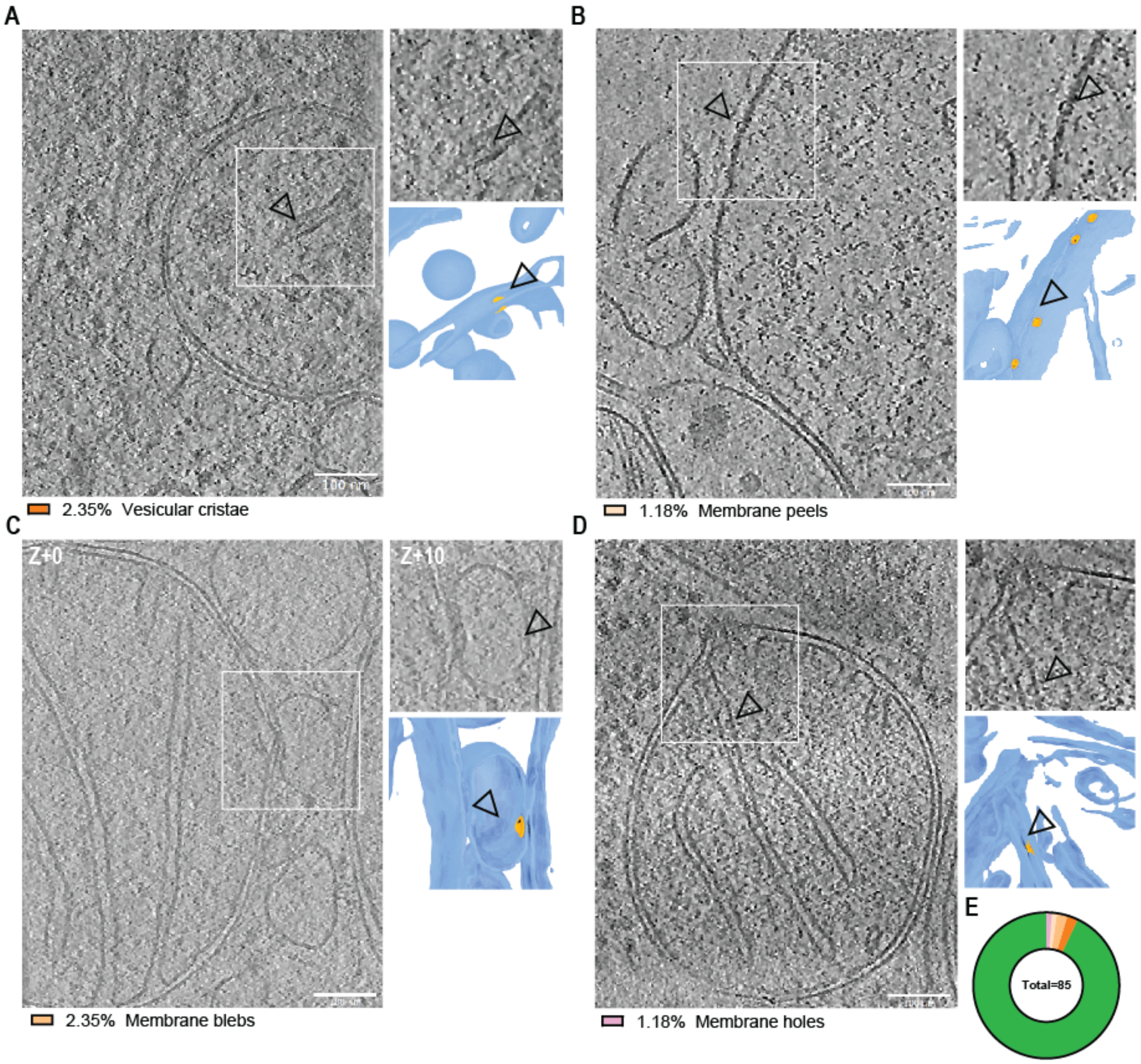

Supplemental Figure 4: Prohibitin is an EM fiducial for studying mitochondrial membrane morphology changes. Back projection of prohibitin particles into raw tomograms identified mitochondria with membrane distortions. (A) A mitochondrial fragment with spherical cristae is juxtaposed by a single tubular crista containing prohibitin (inset). Prohibitin complexes were also identified exposed to the cytosol in mitochondria with outer membrane peels (B and inset). A single layer outer membrane bleb from a mitochondria is identified by a prohibitin complex on the interior of the bleb (C and inset). A double membrane rupture of a mitochondria is shown with prohibitin present in the cristae below the rupture site (D and inset). (E) Comparison of membrane morphologies from each class (n=85 fragments, 79 with no abnormalities).

bioRxiv preprint doi: https://doi.org/10.1101/2025.03.24.645001; this version posted March 25, 2025. The copyright holder for this preprint (which was not certified by peer review) is the author/funder, who has granted bioRxiv a license to display the preprint in perpetuity. It is made available under aCC-BY 4.0 International license.

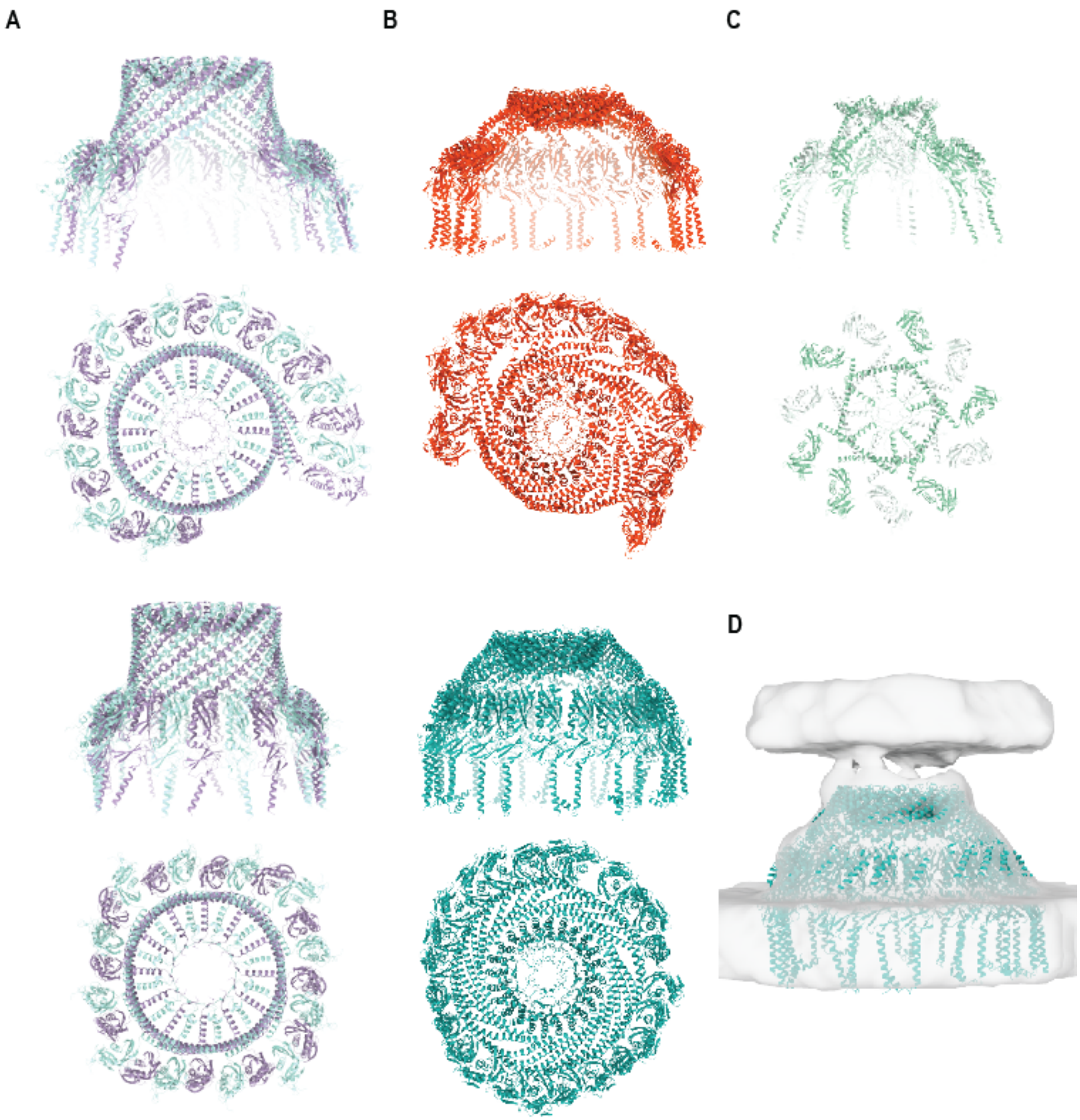

Supplementary Figure 5: Structural comparison of HFLC/K and prohibitin structures. Structures of HFLC/K complex in the open (PDB: 9CZ2) and closed states (PDB: 7VHP) (A). Prohibitin models (open at top, closed at bottom) generated in this study (B). A prohibitin model generated from C11 symmetric processing (PDB: 8RRH) (C). Closed model from this study docked into the density map of putative prohibitin from Chlamydomonas (EMDB-50212) (D).

bioRxiv preprint doi: https://doi.org/10.1101/2025.03.24.645001; this version posted March 25, 2025. The copyright holder for this preprint (which was not certified by peer review) is the author/funder, who has granted bioRxiv a license to display the preprint in perpetuity. It is made available under aCC-BY 4.0 International license.

Table S1. Cryo-electron tomography data collection

Dataset #	1 G3	2 G3	3 G4	4 G2
Grids	Quantifoil gold R2/2	Quantifoil gold R2/2	Quantifoil gold R2/2	Quantifoil gold R2/2
Cell type	U2OS	U2OS	U2OS	U2OS
Cryo-Specimen Freezing	Vitrobot Mark IV	Vitrobot Mark IV	Vitrobot Mark IV	Vitrobot Mark IV
Microscope, Voltage (keV)	Titan Krios G3, 300	Titan Krios G3, 300	Titan Krios G4, 300	Titan Krios G3, 300
Detector	Gatan Quantum K3	Gatan Quantum K3	Falcon i4	Gatan Quantum K3
Energy filter slit width (eV)	25	25	10	20
Electron Exposure ($e^-/Å^2$) dose fractionation	~90	~90	~120	~120
Defocus Range (μm)	-2 μm to -6 μm	-2 μm to -6 μm	-2 μm to -6 μm	-2 μm to -6 μm
Tilt scheme	-60°/+60°, 3°, dose symmetrical (Hagen Scheme)	60°/+60°, 3°, dose symmetrical (Hagen Scheme)	60°/+60°, 3°, dose symmetrical (Hagen Scheme)	60°/+60°, 3°, dose symmetrical (Hagen Scheme)
Movie recording	6-8	6-8	9	6-8
Magnification (times)	43000	43000	64000	42000
Pixel Size (Å/px)	1.05 (Super resolution)	1.05 (Super resolution)	1.965	0.90 (Super resolution)
Tomograms acquired	21	50	47	33

bioRxiv preprint doi: https://doi.org/10.1101/2025.03.24.645001; this version posted March 25, 2025. The copyright holder for this preprint (which was not certified by peer review) is the author/funder, who has granted bioRxiv a license to display the preprint in perpetuity. It is made available under aCC-BY 4.0 International license.

Movie titles

Movie 1: Representative tomogram of an untreated mitochondria.

Movie 2: Representative tomogram of an OA treated mitochondria

Movie 3: Tomogram of a phagophore with putative BLTPs targeting a damaged mitochondrial fragment.

Movie 4: Tomogram showing membranes enveloping an OA treated mitochondrial fragment.

Movie 5: Morph of prohibitin maps and models illustrating differences between the two conformations.
